# Supplementary material for: Exploration of Driver Posture Monitoring Using Pressure Sensors with Lower Resolution
Source: Sensors (Basel). 2021 May 12;21(10):3346. doi: 10.3390/s21103346 (PMC8151731; doi:10.3390/s21103346)
Supplement: Supplementary file 1 [file sensors-21-03346-s001.zip › sensors-1206110-supplementary.pdf]

sensors

MDPI

Table S1. Pressure features extracted from segmented pressure mats.

**Sensing area**	**Contact Area Proportion (CAP)**	**Center of Pressure (COP)**	**Pressure Ratio pair (PR, area 1 / area 2)**
		**Backrest pressure mat**	
{Bi}(i = 1,2, … , 12)	$\frac{\sum_{area} n(i,j) = 1}{N}$ Where $\mathrm{n}(i,j) = 1$ if the cell at position (i,j) within a sensing area on the backrest is occupied, 0 otherwise. $N = 44 \times 42$	The COP of a sensing area in both up-down and left-right directions on the backrest	Bi/B
B			--
B1B2			B1B2/B
B3B4			B3B4/B
B5B6			B5B6/B
B7B8			B7B8/B
B9B10			B11B12/B
B11B12			B11B12/B
B1B5B9			B1B5B9/B
B2B6B10			B2B6B10/B
B3B7B11			B3B7B11/B
B4B8B12			B4B8B12/B
B1B2B3B4			B1B2B3B4/B
B5B6B7B8			B5B6B7B8/B
B9B10B11B12			B9B10B11B12/B
B1B2B5B6B9B10			B1B2B5B6B9B10/B
B3B4B7B8B11B12			B3B4B7B8B11B12/B
		**Seat pan pressure mat**	
{Si}(i = 1,2, … , 8)	$\frac{\sum_{area} n(i,j) = 1}{N}$ Where $\mathrm{n}(i,j) = 1$ if the cell at position (i,j) within a sensing area on the seat pan is occupied, 0 otherwise. $N = 44 \times 42$	The COP of a sensing area in both fore-aft and left-right directions on the seat pan	Si/S
S			--
S1S2			S1S2/S<br>S1S2/S1S2S3S4
S3S4			S3S4/S
S5S6			S5S6/S
S7S8			S7S8/S<br>S7S8/S5S6S7S8
S1S3			S1S3/S<br>S1S3/S1S2S3S4<br>S1S3/S1S3S5S7
S2S4			S2S4/S
S5S7			S5S7/S<br>S5S7/S5S6S7S8
S6S8			S6S8/S<br>S6S8/S2S4S6S8
S1S2S3S4			S1S2S3S4/S
S5S6S7S8			S5S6S7S8/S
S1S3S5S7			S1S3S5S7/S
S2S4S6S8			S2S4S6S8/S
Note. If one sensing area (individual subarea or combine subareas) has no contact with driver's body, the pressure features related to this sensing area will be given NaN.			

Table S2. Best feature combinations used by classifiers RF-trunk, RF-leftFoot and RF-rightFoot. X_COP_U (V) stands for the COP position of area X in left-right (up-down) direction on backrest and fore-aft (left-right) direction on seat pan. X_CAP is the contact proportion within area X. X_Y_PR is referred to as the ratio of the pressure sum between area X and area Y. For each body part, the features are ranked according to their importance estimated by OOB errors.

Important features used by RF-trunk					
ID	Feature	ID	Feature	ID	Feature
1	B_CAP	10	B2_B_PR	19	S5S6S7S8_COP_V
2	B2B6B10_B_PR	11	S_CAP	20	B5B6B7B8_B_PR
3	B5_B_PR	12	S4_S_PR	21	S5S6S7S8_COP_U
4	B1B5B9_B_PR	13	S1S2S3S4_S_PR	22	S_COP_V
5	B_COP_U	14	B6_B_PR	23	S4_COP_V
6	S_COP_U	15	S7S8_S5S6S7S8_PR	24	B11_B_PR
7	B9_B_PR	16	B4B8B12_B_PR	25	B7_B_PR
8	S1S2S3S4_COP_U	17	S5_S_PR	26	S6S8_S2S4S6S8_PR
9	B_COP_V	18	S1_S_PR	27	S8_S_PR
Important features used by RF-leftFoot					
ID	Feature	ID	Feature	ID	Feature
1	S4_CAP	9	S6_COP_V	17	B1B2B3B4_CAP
2	B9B10_CAP	10	B_CAP	18	B6_CAP
3	B10_CAP	11	B5B6_COP_U	19	B1_CAP
4	B4B8B12_B_PR	12	S_CAP	20	B1_COP_U
5	B1B2B5B6B9B10_CAP	13	S3S4_CAP	21	B9_CAP
6	B9B10B11B12_CAP	14	B1_B_PR	22	S7_CAP
7	S2_S_PR	15	S1S3_S1S2S3S4_PR	23	S4_COP_U
8	B5_CAP	16	B7B8_COP_U	24	B5_COP_V
Important features used by RF-rightFoot					
ID	Feature	ID	Feature	ID	Feature
1	S1_S_PR	9	S8_CAP	17	S2S4S6S8_CAP
2	S8_COP_V	10	B10_CAP	18	S2_COP_V
3	B_CAP	11	S6_CAP	19	B3_COP_U
4	S5_COP_V	12	B1_B_PR	20	B2_CAP
5	B9_CAP	13	B5_CAP	21	B6_CAP
6	S5S7_COP_V	14	S7_COP_V	22	B12_B_PR
7	S7S8_COP_V	15	B12_CAP		
8	B11_COP_V	16	B1_CAP		

© 2021 by the authors. Submitted for possible open access publication under the terms and conditions of the Creative Commons Attribution (CC BY) license (http://creativecommons.org/licenses/by/4.0/).
